# Supplementary material for: Middle-aged individuals may be in a perpetual state of H3N2 influenza virus susceptibility
Source: Nat Commun. 2020 Sep 11;11:4566. doi: 10.1038/s41467-020-18465-x (PMC7486384; doi:10.1038/s41467-020-18465-x)
Supplement: Supplementary file 1 — Supplementary Information [file 41467_2020_18465_MOESM1_ESM.pdf]

Supplementary Table 1. Number of serum samples per age group.

Age group (years)	Number of samples (n)
1-2	47
3-10	81
11-20	22
21-30	32
31-40	36
41-50	27
51-60	31
61-70	34*
71-90	42

* Sera from 2 participants in this age group were not tested in ELLA because of insufficient sample volume.

Supplementary Table 2. Comparisons of FRNT titers between age groups.

3c2.A									
	1-2	3-10	11-20	21-30	31-40	41-50	51-60	61-70	71-90
1-2		1.9 [1.2,2.5]<br>(< 0.0007)	0.6 [-0.2,1.3]<br>(0.057)	0.7 [0.0,1.4]<br>(0.016)	0.0 [-0.6,0.6]<br>(0.459)	-0.5 [-1.1,0.1]<br>(0.945)	-0.2 [-0.9,0.5]<br>(0.723)	0.3 [-0.5,1.0]<br>(0.249)	0.5 [-0.2,1.2]<br>(0.067)
3-10	-1.9 [-2.5,-1.2]<br>(1.000)		-1.3 [-2.0,-0.6]<br>(1.000)	-1.2 [-1.8,-0.5]<br>(1.000)	-1.8 [-2.4,-1.3]<br>(1.000)	-2.3 [-2.9,-1.8]<br>(1.000)	-2.1 [-2.7,-1.4]<br>(1.000)	-1.6 [-2.3,-0.9]<br>(1.000)	-1.3 [-2.0,-0.7]<br>(1.000)
11-20	-0.6 [-1.3,0.2]<br>(0.943)	1.3 [0.6,2.0]<br>(< 0.0007)		0.1 [-0.6,0.8]<br>(0.384)	-0.6 [-1.2,0.1]<br>(0.949)	-1.1 [-1.7,-0.4]<br>(1.000)	-0.8 [-1.5,0.0]<br>(0.985)	-0.3 [-1.1,0.5]<br>(0.801)	-0.1 [-0.8,0.7]<br>(0.569)
21-30	-0.7 [-1.4,0.0]<br>(0.984)	1.2 [0.5,1.8]<br>(< 0.0007)	-0.1 [-0.8,0.6]<br>(0.616)		-0.7 [-1.2,-0.1]<br>(0.988)	-1.2 [-1.8,-0.6]<br>(1.000)	-0.9 [-1.6,-0.2]<br>(0.997)	-0.5 [-1.1,0.3]<br>(0.897)	-0.2 [-0.8,0.5]<br>(0.692)
31-40	0.0 [-0.6,0.6]<br>(0.541)	1.8 [1.3,2.4]<br>(< 0.0007)	0.6 [-0.1,1.2]<br>(0.051)	0.7 [0.1,1.2]<br>(0.012)		-0.5 [-1.0,0.0]<br>(0.980)	-0.2 [-0.8,0.4]<br>(0.780)	0.2 [-0.4,0.9]<br>(0.257)	0.5 [-0.1,1.1]<br>(0.063)
41-50	0.5 [-0.1,1.1]<br>(0.055)	2.3 [1.8,2.9] (<br>< 0.0007)	1.1 [0.4,1.7]<br>(< 0.0007)	1.2 [0.6,1.8]<br>(< 0.0007)	0.5 [0.0,1.0]<br>(0.020)		0.3 [-0.3,0.9]<br>(0.170)	0.7 [0.1,1.4]<br>(0.015)	1.0 [0.4,1.6]<br>(0.001)
51-60	0.2 [-0.5,0.9]<br>(0.277)	2.1 [1.4,2.7]<br>(< 0.0007)	0.8 [0.0,1.5]<br>(0.015)	0.9 [0.2,1.6]<br>(0.003)	0.2 [-0.4,0.8]<br>(0.220)	-0.3 [-0.9,0.3]<br>(0.830)		0.5 [-0.3,1.2]<br>(0.112)	0.7 [0.0,1.4]<br>(0.019)
61-70	-0.3 [-1.0,0.5]<br>(0.751)	1.6 [0.9,2.3]<br>(< 0.0007)	0.3 [-0.5,1.1]<br>(0.201)	0.5 [-0.3,1.1]<br>(0.103)	-0.2 [-0.9,0.4]<br>(0.743)	-0.7 [-1.4,-0.1]<br>(0.985)	-0.5 [-1.2,0.3]<br>(0.888)		0.3 [-0.5,1.0]<br>(0.243)
71-90	-0.5 [-1.2,0.2]<br>(0.933)	1.3 [0.7,2.0]<br>(< 0.0007)	0.1 [-0.7,0.8]<br>(0.431)	0.2 [-0.5,0.8]<br>(0.309)	-0.5 [-1.1,0.1]<br>(0.938)	-1.0 [-1.6,-0.4]<br>(0.999)	-0.7 [-1.4,0.0]<br>(0.981)	-0.3 [-1.0,0.5]<br>(0.757)	
3c2.A2									
	1-2	3-10	11-20	21-30	31-40	41-50	51-60	61-70	71-90
1-2		1.9 [1.2,2.5]<br>(< 0.0007)	0.4 [-0.4,1.2]<br>(0.168)	-0.1 [-0.8,0.5]<br>(0.654)	-0.6 [-1.2,-0.1]<br>(0.981)	-0.8 [-1.4,-0.3]<br>(0.997)	-0.6 [-1.3,0.1]<br>(0.942)	-0.5 [-1.2,0.1]<br>(0.939)	-0.3 [-1.0,0.3]<br>(0.844)
3-10	-1.9 [-2.5,-1.2]<br>(1.000)		-1.5 [-2.2,-0.7]<br>(1.000)	-2.0 [-2.6,-1.4]<br>(1.000)	-2.5 [-3.0,-2.0]<br>(1.000)	-2.7 [-3.2,-2.2]<br>(1.000)	-2.4 [-3.0,-1.8]<br>(1.000)	-2.4 [-3.0,-1.8]<br>(1.000)	-2.2[-2.8,-1.6]<br>(1.000)
11-20	-0.4 [-1.2,0.4]<br>(0.833)	1.5 [0.7,2.2]<br>(< 0.0007)		-0.5 [-1.3,0.2]<br>(0.918)	-1.0 [-1.7,-0.4]<br>(0.999)	-1.2 [-1.9,-0.6]<br>(1.000)	-1.0 [-1.7,-0.2]<br>(0.993)	-0.9 [-1.6,-0.2]<br>(0.992)	-0.7 [-1.4,0.0]<br>(0.977)
21-30	0.1 [-0.5,0.8]<br>(0.348)	2.0 [1.4,2.6]<br>(< 0.0007)	0.5 [-0.2,1.3]<br>(0.083)		-0.5 [-1.0,0.0]<br>(0.969)	-0.7 [-1.2,-0.2]<br>(0.997)	-0.4 [-1.0,0.2]<br>(0.908)	-0.4 [-1.0,0.2]<br>(0.896	-0.2 [-0.8,0.4]<br>(0.739)
31-40	0.6 [0.1,1.2]<br>(0.019)	2.5 [2.0,3.0]<br>(< 0.0007)	1.0 [0.4,1.7]<br>(0.001)	0.5 [0.0,1.0]<br>(0.032)		-0.2 [-0.6,0.1]<br>(0.887)	0.1 [-0.4,0.6]<br>(0.364)	0.1 [-0.3,0.6]<br>(0.303)	0.3 [-0.1,0.7]<br>(0.078)
41-50	0.8 [0.3,1.4]<br>(0.003)	2.7 [2.2,3.2]<br>(< 0.0007)	1.2 [0.6,1.9]<br>(< 0.0007)	0.7 [0.2,1.2]<br>(0.003)	0.2 [-0.1,0.6]<br>(0.118)		0.3 [-0.2,0.8]<br>(0.136)	0.3 [-0.1,0.8]<br>(0.086)	0.5 [0.1,0.9]<br>(0.008)
51-60	0.6 [-0.1,1.3]<br>(0.058)	2.4 [1.8,3.0]<br>(< 0.0007)	1.0 [0.2,1.7]<br>(0.007)	0.4 [-0.2,1.0]<br>(0.092)	-0.1 [-0.6,0.4]<br>(0.636)	-0.3 [-0.8,0.2]<br>(0.864)		0.0 [-0.6,0.6]<br>(0.463)	0.2 [-0.4,0.8]<br>(0.214)
61-70	0.5 [-0.1,1.2]<br>(0.061)	2.4 [1.8,3.0]<br>(< 0.0007)	0.9 [0.2,1.6]<br>(0.008)	0.4 [-0.2,1.0]<br>(0.105)	-0.1 [-0.6,0.3]<br>(0.700)	-0.3 [-0.8,0.1]<br>(0.914)	0.0 [-0.6,0.6]<br>(0.537)		0.2 [-0.3,0.7]<br>(0.235)
71-90	0.3 [-0.3,1.0]<br>(0.157)	2.2 [1.6,2.8]<br>(< 0.0007)	0.7 [0.0,1.4]<br>(0.024)	0.2 [-0.4,0.8]<br>(0.263)	-0.3 [-0.7,0.1]<br>(0.925)	-0.5 [-0.9,-0.1]<br>(0.993)	-0.2 [-0.8,0.4]<br>(0.786)	-0.2 [-0.7,0.3]<br>(0.765)	

Each cell shows a titer difference (column minus row) between age groups (in years). P-values for the differences were calculated using bootstrapping and are shown in parentheses. After applying Bonferroni correction for multiple comparisons, $p<0.0007$ indicates the difference is significantly greater than 0.

Supplementary Table 3. The estimates of the effects of imprinting, vaccine coverage effect, and virus specific effect.

Model	Variable	Estimate (standard error)	p	∆AIC
Model 1				0
	Imprinting to H3	0.17 (0.30)	0.58	
	Amino acid similarity between first H3 virus in life and a test virus	3.45 (0.38)	<0.001	
	Vaccine coverage	3.86 (0.56)	<0.001	
	Virus effect for 3c2.A2	-0.23 (0.12)	0.05	
Model 2 (with linear age effect)				1.4
	Imprinting to H3	0.33 (0.36)	0.37	
	Amino acid similarity between first H3 virus in life and a test virus	3.57 (0.41)	<0.001	
	Age	0.003 (0.003)	0.44	
	Vaccine coverage	4.19 (0.71)	<0.001	
	Virus effect for 3c2.A2	-0.23 (0.12)	0.06	
Model 3 (with age group effect for <18)				1.9
	Imprinting to H3	0.12 (0.32)	0.71	
	Amino acid similarity between first H3 virus in life and a test virus	3.37 (0.44)	<0.001	
	Age group <18	0.08 (0.24)	0.73	
	Vaccine coverage	3.60 (0.94)	<0.001	
	Virus effect for 3c2.A2	-0.23 (0.12)	0.05	

Linear regression is performed to fit each model uding glm function in R (version 3.4.2). Akaike information criterion (AIC) is used for model comparison.

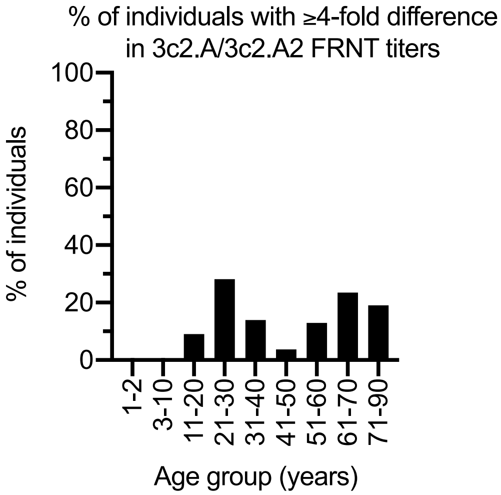

Supplementary Figure 1. Percentage of individuals with a ≥4-difference in neutralizing antibody titers as measured in FRNT in Figure 1. Source data are provided as a Source Data file.
