# Supplementary material for: Middle-aged individuals may be in a perpetual state of H3N2 influenza virus susceptibility
Source: Nat Commun. 2020 Sep 11;11:4566. doi: 10.1038/s41467-020-18465-x (PMC7486384; doi:10.1038/s41467-020-18465-x)
Supplement: Supplementary file 2 — Reporting Summary [file 41467_2020_18465_MOESM2_ESM.pdf]

nature research

Corresponding author(s): Scott E. Hensley

Last updated by author(s): Aug 3, 2020

Reporting Summary

Nature Research wishes to improve the reproducibility of the work that we publish. This form provides structure for consistency and transparency in reporting. For further information on Nature Research policies, see Authors & Referees and the Editorial Policy Checklist .

Statistics

For all statistical analyses, confirm that the following items are present in the figure legend, table legend, main text, or Methods section.

n/a	Confirmed	
☐	☒	The exact sample size (n) for each experimental group/condition, given as a discrete number and unit of measurement
☐	☒	A statement on whether measurements were taken from distinct samples or whether the same sample was measured repeatedly
☐	☒	The statistical test(s) used AND whether they are one- or two-sided<br>*Only common tests should be described solely by name; describe more complex techniques in the Methods section.*
☐	☒	A description of all covariates tested
☐	☒	A description of any assumptions or corrections, such as tests of normality and adjustment for multiple comparisons
☐	☒	A full description of the statistical parameters including central tendency (e.g. means) or other basic estimates (e.g. regression coefficient) AND variation (e.g. standard deviation) or associated estimates of uncertainty (e.g. confidence intervals)
☐	☒	For null hypothesis testing, the test statistic (e.g. F, t, r) with confidence intervals, effect sizes, degrees of freedom and P value noted<br>*Give P values as exact values whenever suitable.*
☒	☐	For Bayesian analysis, information on the choice of priors and Markov chain Monte Carlo settings
☒	☐	For hierarchical and complex designs, identification of the appropriate level for tests and full reporting of outcomes
☒	☐	Estimates of effect sizes (e.g. Cohen's d, Pearson's r), indicating how they were calculated

Our web collection on statistics for biologists contains articles on many of the points above.

Software and code

Policy information about availability of computer code

Data collection	No software was used for data collection.
Data analysis	GraphPad Prism version 7, R version 3.4.2

For manuscripts utilizing custom algorithms or software that are central to the research but not yet described in published literature, software must be made available to editors/reviewers. We strongly encourage code deposition in a community repository (e.g. GitHub). See the Nature Research guidelines for submitting code & software for further information.

Data

Policy information about availability of data

All manuscripts must include a data availability statement. This statement should provide the following information, where applicable:

- Accession codes, unique identifiers, or web links for publicly available datasets
- A list of figures that have associated raw data
- A description of any restrictions on data availability

Source data for Figures 1-3 and for Supplementary Figures 1 and 2 are provided in the Source Data file. The sequences used in Figure 4A are available via GenBank (accession codes EF409245, CY113261, U97740, AJ311466, EU199250 and MH586372).

Field-specific reporting

Please select the one below that is the best fit for your research. If you are not sure, read the appropriate sections before making your selection.

☒ Life sciences ☐ Behavioural & social sciences ☐ Ecological, evolutionary & environmental sciences

Life sciences study design

All studies must disclose on these points even when the disclosure is negative.

Sample size	No sample size calculations were performed. Sera from the Children's Hospital of Philadelphia were leftover from lead testing and all available sera collected between May 1, 2017 and August 31, 2017 were used in this study.<br>Sera from the Hospital of the University of Pennsylvania were also collected between May 1, 2017 and August 31, 2017. Sera were selected based on age, selecting no more than 40 sera per 10-year age bin to have a wide age range.<br>Sera collected in 2014-2015 in Ann Arbor, Michigan, and in 2016-2017 in Rochester, New York, were included based on sample availability.
Data exclusions	For the samples collected at the Hospital of the University of Pennsylvania, some pre-established exclusion criteria were used to exclude samples from donors who have an underlying condition that could affect antibody levels in general. Samples from donors who had a pregnancy reported during the last 9 months, who had a medical history of cancer or organ transplantation, or who had a reported infectious disease within the previous 28 days were excluded.<br>Sera from 2 participants in the age group 61-70 were not tested in ELLA due to insufficient sample volume.<br>From the sera collected in 2014-2015 in Michigan, Ann Arbor, only sera from middle-aged adults born in 1963-1979 (n=14) were included for analysis.
Replication	Replication was not performed for the large serological screen. Testing a larger sample size was preferred over testing a smaller sample size in duplicate. When other samples were tested in duplicate on different days, no more than 2-fold differences between measurements were observed.
Randomization	Randomization was not applicable for this retrospective study. Samples were included based on sample availability with very limited information on the subjects.
Blinding	Blinding was not applicable. All samples in this study were de-identified.

Reporting for specific materials, systems and methods

We require information from authors about some types of materials, experimental systems and methods used in many studies. Here, indicate whether each material, system or method listed is relevant to your study. If you are not sure if a list item applies to your research, read the appropriate section before selecting a response.

Materials & experimental systems

n/a	Involved in the study	
☐	☒	Antibodies
☐	☒	Eukaryotic cell lines
☒	☐	Palaeontology
☒	☐	Animals and other organisms
☐	☒	Human research participants
☒	☐	Clinical data

Methods

n/a	Involved in the study	
☒	☐	ChIP-seq
☒	☐	Flow cytometry
☒	☐	MRI-based neuroimaging

Antibodies

Antibodies used	Monoclonal antibody 041-10047-1C04 was isolated following vaccination with the 2010/2011 influenza vaccine (doi: 10.1016/j.celrep.2019.11.084).<br><br>Anti-NP monoclonal antibody IC5-1B7 (product number NR-43899; BEI Reagent Resources)<br><br>Anti-mouse peroxidase-conjugated secondary antibody (product number 855563; MP Biomedicals)<br><br>Peroxidase AffiniPure F(ab')$_2$ Fragment Goat Anti-Human IgG, Fcγ Fragment Specific (product number 109-036-098; Jackson ImmunoResearch Laboratories, Inc)
Validation	IC5-1B7 was verified to be NP-specific via western blot (BEI Reagent Resources web page). The anti-mouse antibody and anti-human antibody were both tested for purity by immunoelectrophoresis.<br><br>Antibodies have been used for FRNT and ELISA in other publications:<br>- doi: 10.1093/cid/ciz996<br>- doi: 10.1073/pnas.1712377114<br>- doi: 10.1128/JVI.02134-18<br>- doi: 10.1016/j.celrep.2019.11.084

Eukaryotic cell lines

Policy information about cell lines

Cell line source(s)	MDCK-SIAT1 cells were obtained from Fred Hutchinson Cancer Center; 293T cells were obtained from the NIH; 293F cells were obtained from ThermoFisher
Authentication	The MDCK-SIAT1 cell line was not authenticated but these cells are routinely used in our laboratory for influenza virus assays.
Mycoplasma contamination	Cell lines tested negative for mycoplasma contamination.
Commonly misidentified lines (See ICLAC register)	No commonly misidentified cell lines were used in the study.

Human research participants

Policy information about studies involving human research participants

Population characteristics	Serum samples were collected at the Children's Hospital of Philadelphia from children (age 1-17, gender unknown) and at the Hospital of the University of Pennsylvania from adults (age 18-90, 107 males and 105 females). Because gender information was not available for sera collected at the Children's Hospital of Philadelphia, this information was not included in the manuscript. All sera collected in 2014-2015 in Ann Arbor, Michigan, were from individuals born between 1963-1979. Sera collected in 2016-2017 in Rochester, New York, were from individuals aged 14-55 years.
Recruitment	De-identified serum samples collected in the summer of 2017 for lead testing at the Children's Hospital of Philadelphia and sera collected at the Hospital of the University of Pennsylvania were used in this study. Recruitment for the Household Influenza Vaccine Effectiveness study in Ann Arbor, Michigan has been previously described (Malosh et al., Vaccine 2014). Samples from Rochester, New York, were from individuals who were recruited when they had an acute influenza virus infection in 2016-2017. Vaccination uptake in those cohorts might be higher than the vaccination uptake in the general population.
Ethics oversight	All cohorts involving human samples included in this study were approved by the Institutional Review Boards of the University of Michigan, University of Rochester, and the University of Pennsylvania

Note that full information on the approval of the study protocol must also be provided in the manuscript.
